# Epulis

**Published:** 1908-08-22

**Authors:** 


### EPULIS.

Epulides are more often sarcomatous than not, though the type of the sarcoma is relatively benign—giant-celled sarcoma predominating in all the published statistics upon the subject. The following records of the microscopical findings in thirty-one cases at Tubingen are due to Dr. R. Kühner, and they represent the relative frequency of the different kinds of epulides fairly well:

| | |
|---|---|
| Giant-celled Sarcoma occurred in ... | 20 cases |
| Fibro-sarcoma occurred in ... ... | 4 cases |
| Spindle-celled Sarcoma occurred in ... | 2 cases |
| Fibroma occurred in ... ... ... | 2 cases |
| Osteo-fibro-sarcoma occurred in ... | 1 case |
| Osteoma occurred in ... ... ... | 1 case |
| Granuloma occurred in ... ... | 1 case |

This preponderance of sarcoma agrees well with the fact that epulis is a disease of the comparatively young. It may arise in boys or girls; the average age in a collection of ninety cases was twenty-six years, the mean for male patients being eighteen years and for females thirty-one.

The tumours look so innocent at first that one might be inclined to treat them by simply snipping them off with scissors. The result in almost every such case is recurrence, with increased difficulty in eradicating the growth at a subsequent operation. When, on the other hand, the initial operation is radical, including removal of a tooth or teeth if necessary, and a part of the alveolar process of the jaw in any case, complete cure results in most instances. Radical excision reduces the proportion of recurrences to about 12 per cent. If the tumour is left to itself, death is likely to result in one of three ways—from general sarcomatosis, from fœtid ulceration and sepsis, or from hæmorrhage originating in the epulis. Spontaneous cure is not unknown; it is impossible to say for certain whether the epulis was sarcomatous or not in any such instances, but it seems probable that the lesion in the cases which get well by themselves is more likely granulomatous; simple granuloma occurs in about one case in thirty.

It is a remarkable fact that epulides are far more common in patients of the hospital class than they are amongst those of higher rank in life. This supports the view that lack of care of the mouth and teeth is a potent factor in predisposing to the disease, and is yet another reason for urging all patients to have their teeth kept in good order.

